# Personalized Hemoglobin A1c Shows Better Correlation with Mean Glucose than Laboratory Hemoglobin A1c in Ugandan Youth with Type 1 Diabetes, but Mean Glucose Is Not Clinically Useful in This Population Due to Extreme Glucose Variability

**DOI:** 10.1089/dia.2024.0537

**Published:** 2025-07-29

**Authors:** Thereza Piloya-Were, Catherine Nyangabayaki, Timothy C. Dunn, Daniel Malinga, Jemima Nambooze, Elizabeth Pappenfus, Lin Zhang, Anila Bindal, Shannon Beasley, Muna Sunni, Brandon M. Nathan, Sandy Liu, Antoinette Moran

**Affiliations:** 1 Department of Pediatrics, Makerere University College of Health Sciences, Kampala, Uganda.; 2 Department of Pediatrics, St. Francis Hospital, Kampala, Uganda.; 3 Abbott Diabetes Care, Alameda, California, USA.; 4 Division of Pediatric Endocrinology and Diabetes, Department of Pediatrics, School of Medicine, University of Minnesota, Minneapolis, Minnesota, USA.; 5 Division of Biostatistics, School of Public Health, University of Minnesota, Minneapolis, Minnesota, USA.

**Keywords:** apparent glycation ratio, personalized A1c, racial differences, A1c-MG variability, kinetic modeling

## Abstract

**Introduction:**

Continuous glucose monitoring (CGM) is unaffordable in sub-Saharan Africa, and providers rely heavily on hemoglobin A1c (A1c) to guide insulin adjustment. The relationship between A1c and mean glucose (MG) varies between individuals and populations. We assessed this relationship in Ugandan youth of age 4–26 years with type 1 diabetes, and evaluated whether calculation of the personalized A1c (pA1c), which only requires a brief initial sensor wear, is clinically useful.

**Materials and Methods:**

CGM data were averaged across three blinded sensor wears (31–41 days). We calculated individual apparent glycation ratios using A1c after the second sensor, and applied these to A1cs collected after the third sensor to determine pA1c. Participants were evaluated for clinical factors that influence red blood cell (RBC) lifespan (malaria, G6PD deficiency, sickle-cell trait, hemolysis, iron deficiency).

**Results:**

Patients across the A1c spectrum experienced substantial time in both hyper- and hypoglycemia; average coefficient of variation was 44%. MG was >250 mg/dL (13.9 mmol/L) in 50% of participants, and 55% of participants spent ≥4% time with glucose <70 mg/dL (3.9 mmol/L). There was considerable variability in the A1c-MG relationship. The pA1c more accurately represented MG by significantly reducing variation in this relationship (*R*^2^ = 0.84 vs. 0.40; *r* = 0.92 vs. 0.63), but MG is not useful in individuals with the wide glucose fluctuations seen in this population. Clinical factors did not impact the A1c-MG relationship.

**Conclusions:**

Neither the measured A1c nor the calculated pA1c provided reliable guidance for insulin adjustment in this population. No matter how accurately MG is measured or estimated, it is just an average, with limited clinical application in individuals with wide glycemic variation. These measures cannot replace the information available from CGM about glycemic excursion, daily glucose patterns, or percent time in various glucose ranges. Our data suggest that it is essential to find a way to make CGM at least periodically affordable in low-resource settings.

## Introduction

Since the 1990s, hemoglobin A1c (A1c) has been considered the test of choice for assessment of glycemic control because of its demonstrated association with diabetes complications. While it is commonly considered a proxy for the mean glucose (MG) concentration over the previous 8–12 weeks,^1^ there is significant variability in the relationship between A1c and MG both in populations and in individuals.^2–9^ This is particularly apparent now that MG can be more accurately assessed using continuous glucose monitoring (CGM).

The relationship between A1c and MG is important because patients and health care providers use these metrics to make medication decisions as they strive to achieve optimal blood glucose control. If assumptions about A1c levels are inaccurate, the prescribed insulin dose may be inappropriately increased or decreased. This has become less of an issue in well-resourced settings where CGM is widely available and can provide comprehensive glycemic data, although even in wealthy nations there is socioeconomic and racial disparity in CGM availability.^10,11^ The A1c-MG relationship may be particularly relevant in low/low-middle income nations where care providers rely heavily on A1c levels to guide insulin adjustment. In these countries, CGM technology is inaccessible to people with diabetes, and furthermore, they are rarely able to obtain more than two to three test strips a day for self-monitoring of blood glucose (SMBG) levels.^12^ Ministries of Health are more focused on larger population health issues, with few resources devoted to support of type 1 diabetes (T1D). Point-of-care (POC) A1c testing is commonly available and free to patients because of internationally donated machines, while laboratory-measured A1c may not be affordable.

Following a 2019 pilot study performed in Ugandan and Kenyan youth with T1D who wore a single CGM sensor for 4–15 days,^12^ a secondary analysis comparing those data with other reported populations found that A1c levels consistently overestimated MG concentrations.^9^ This approach did not consider the high individual variability that was found in the A1c-MG relationship. Xu et al. developed a calculation to more accurately characterize MG concentrations relative to A1c levels by accounting for individual differences in RBC characteristics.^8,13^ A brief period of sensor wear is required to calculate a person’s apparent glycation ratio (AGR), which can then be used to adjust future A1c measurements to estimate a personalized A1c (pA1c) level with better concordance with MG.

The current observational study was performed in a cohort of Ugandan youth of age 4–26 years with T1D, to further characterize the relationship between MG and both laboratory and point-of-care (POC) A1c levels, and to determine if the pA1c more closely correlates with MG. While chronic CGM use is prohibitively expensive in this setting, we hypothesized that using just two CGM wears for AGR calculation would allow estimation of pA1c levels that would better reflect true MG, and could thus be utilized at subsequent clinic visits as a practical, cost-effective way to provide clinically relevant information.

Participants were also evaluated for regional factors that might influence RBC lifespan such as malaria, G6PD deficiency, sickle-cell trait, hemolysis, and iron deficiency, and whether there were any associations between these and A1c-MG concordance.

## Materials and Methods

### T1D management in Uganda

Ugandan patients with childhood-onset diabetes are commonly seen in the pediatric diabetes clinics monthly through age 26 years. T1D is diagnosed clinically, since autoantibody testing is neither affordable nor readily available. Children commonly present in diabetic ketoacidosis (DKA) and with significant wasting, reducing the risk of misclassifying type 2 diabetes (T2D). Private health insurance is rare, and both insulin and supplies for SMBG two to three times per day are obtained primarily by donation from the nonprofit organizations, Changing Diabetes in Children (CDiC®, Novo Nordisk Global) and Life For a Child (Diabetes Australia). They also donate POC-A1c machines and associated supplies to the clinics.

T.P.-W. is a pediatric endocrinologist who received her training through the Pediatric Endocrinology Training Center for Africa (PETCA) fellowship program in Nairobi, Kenya. C.N. is a pediatrician with years of experience running a pediatric diabetes program. They are responsible for training the medical officers and nurses on their teams, with additional education provided by the CDiC® program and the University of Minnesota.

Analog insulin availability is limited, and the majority of patients are managed with multiple daily injections of NPH and regular insulin or, occasionally, with premixed insulin Mixtard (Novo Nordisk, 70% NPH and 30% regular insulin). Insulin glargine, used in combination with regular insulin, has recently become available for some children. Free insulin is dispensed monthly in clinic, and in the absence of a refrigerator, East African Diabetes Study Group Guidelines recommend that it be stored in a clean container in a cupboard at room temperature.^14^ A1c does not differ between patients who store their insulin in this manner and those who have access to refrigeration.^15,16^ Currently, use of CGM systems or insulin pumps is almost nonexistent in Uganda.

### Participants

This study was conducted in Kampala, Uganda, at the two major centers caring for children and youth with diabetes. Mulago Hospital (Mulago) is the National Referral Hospital and the teaching hospital for the Makerere University College of Health Sciences; patients there often come from poor socioeconomic circumstances. St. Francis Hospital, Nsambya (Nsambya), is a nonprofit Catholic mission hospital; patients are sometimes of modestly higher socioeconomic status than at Mulago. Participants aged 4–26 years with T1D of at least a year duration and whose baseline A1c was 7.5%–13.5% (58–124 mmol/mol) were recruited from their clinics by the local diabetes teams at Mulago and Nsambya. Consent was obtained for those ≥18 years, parental consent for those <18 years, and assent for those ≥ 8 years. Consent forms were available in both English and Luganda, and bilingual study coordinators assisted with the consent process. The protocol and the consent forms were approved by both the University of Minnesota and the Mulago Hospital IRBs.

### Overall study design

The study was initiated by A.M., University of Minnesota (UMN), and T.P.-W., Makerere University, Uganda. It was funded by a grant from Abbott Diabetes, Alameda, California. The role of the UMN team was to train the local Ugandan investigators, to perform study monitoring, and to ensure that the trial was conducted and data were generated, documented, and reported in compliance with the protocol, good clinical practice, and regulatory requirements. The study was conducted on site in Kampala, Uganda, under the direct supervision of the Ugandan Study PI T.P.-W., and the Nsambya Site PI, C.N.

The study used the Libre Pro Flash Glucose Monitoring system (Abbott Diabetes, Alameda, California). The device’s sensor measures interstitial glucose concentrations in the range of 40–500 mg/dL (2.2–27.8 mmol/L) every 15 min for up to 14 days of wear. A blinded system was chosen to avoid influencing patient and provider behavior to reduce glucose inconsistency across three sensor wears. Neither the patient nor the research team had access to sensor data until the patient had completed the study, at which time the data were available for insulin adjustment. During the study, patient care was delivered according to the local standard, with patients performing SMBG three times per day.

Sensors were placed on participants in clinic by the medical team. As shown in Supplementary Figure S1, each participant wore three consecutive sensors, each for 10–14 days of duration (depending on sensor survival and patient/clinic schedules), with no more than 48 h between sensor wears. If there was not at least 10 days of sensor data or if there were >48 h between sensor wears, the patient was offered the opportunity to start over. Only one participant was unable to complete the CGM protocol. A1c levels were measured both by POC and the laboratory at the end of each sensor wear. MG concentrations over the first two sensor wears and the A1c level collected after the second sensor wear were used for determination of each individual’s AGR, as described below. All other assessments were performed after three sensor wears. At baseline, participant demographic characteristics were obtained, and blood was sent for laboratory assessment of clinical factors that might influence A1c, as listed below.

### CGM assessments

Average CGM-based glucose and coefficient of variation (CV) were calculated across the consecutive sensor wears. Times in glucose ranges were assessed according to international standards.^17^ For the cohort, this included percent time <54 mg/dL (3.0 mmol/L), <70 mg/dL (3.9 mmol/L), 70–180 mg/dL (3.9–10.0 mmol/L), >180 mg/dL (10.0 mmol/L), and >250 mg/dL (13.9 mmol/L).

### Blood sample assessment

Both hospitals had BioHermes Glycohemoglobin Test Kit analyzers (EZ 2.0, China) for POC A1c measurement. This system is NGSP and IFCC double certified. Analyzer quality controls were run on each day of use. Laboratory A1c was determined by a fully automated COBAS 311 analyzer, based on the turbidimetric inhibition immunoassay using whole blood.

Complete blood count (CBC) with differential was determined on a Mindray Hematology Analyzer (BC-5380), China. A peripheral smear was performed by the same hematopathologist. Other laboratory measurements included reticulocyte count (Horiba Hematology Analyzer, Kyoto, Japan), hemoglobin electrophoresis (Minicap flex piercing method, Evry, France), lactate dehydrogenase (LDH) (Cobas Integra 400 plus*,* Roche, Switzerland), ferritin (Maglumi 800, Snibe, China), total iron binding capacity (DAR 800, Diagnostic Automation, California, USA), and G6PD (the Randox Rx Monza instrument using the Randox G6PD kit).

MBN Clinical Laboratories, an AABB-accredited clinical laboratory near Mulago Hospital in Kampala, performed all sample testing. Samples from each hospital were packaged in a cooler with ice packs and were transported to the laboratory within 4 h of blood draw. The study’s two hospital clinics are each within 5 km of the laboratory.

### Calculation of the AGR and the pA1c

The definition and assumptions used for calculation of an individual’s AGR and use of the AGR to calculate a pA1c for that patient at subsequent visits have been described in detail elsewhere.^8,13,18^ Briefly, the AGR reflects an individual’s particular glycation tendency, and once determined it can be used to adjust measured A1c levels to more closely reflect that person’s MG concentration. AGR is most accurate in settings of glucose stability over the approximately 3-month lifespan of the RBC. Once it has been calculated, the AGR is assumed to be relatively constant for that person as long as the RBC physiology remains stable. It can be applied to future A1c levels to create a pA1c that more accurately reflects the MG at that time, to help guide clinical management. This study used the MG calculated over the first two sensor wears, the laboratory A1c at the end the two sensor wears, and a universal RBC binding affinity of glucose on transport-1 Michaelis constant K_m_ (472 mg/dL), to calculate each person’s AGR (ml/g), using the following formula:

$$\mathrm{AGR}\;=\;\frac{{\left[\mathrm{MG}\right]}^{-1}+K_{M}^{-1}}{{100\;*\;[A1c]}^{-1}\;-1}\;*\;10^{5}\;\mathrm{PGR}\;=\;\left({\mathrm{MG}}^{-1}\;+\;K_{M}^{-1}\right)\;*\;{\left(100\;*{\;\mathrm{HbA}}_{1c}^{-1}\;-1\right)}^{-1}\;*\;10^{5}$$

After the third sensor wear and laboratory A1c measurement, that A1c, the previously determined AGR, and a standard RBC lifespan glycation rate constant of 65.1 mL/mg were used to calculate the pA1c for that visit:

$$\mathrm{pA}1C=\frac{100}{1+\frac{\mathrm{AGR}}{65.1}\left(\frac{100}{\left[A1c\right]}-1\right)}\;\mathrm{pA}1C=100\;*\;{\left(1+\frac{\mathrm{PGR}}{{\mathrm{PGR}}_{\mathrm{ref}}}\left(\frac{100}{{\mathrm{HbA}}_{1c}}-1\right)\right)}^{-1}$$

### Sample size and power calculations

Pilot data obtained in a similar population^12^ provided an estimate of 0.52 for the correlation coefficient between A1c and MG. A sample size of 60 was determined to have >95% power to detect the correlations between A1c and MG for the estimated value, with adjustment for the MG obtained from the three CGM sensor wears on each participant.

### Statistical analyses

Two-sample *t*-tests were used to compare the means of metabolic variables during the three sensor wears between children (4–17 years) versus young adults (18–26 years). Simple linear regression analyses were conducted to evaluate the association between MG and laboratory A1c, POC A1c, and pA1c with *R*^2^ calculated. In addition, multiple linear regression analyses were performed to examine the association between MG and A1c, after adjusting for demographical and clinical covariates, including age group, sex, Tanner stage, income level, hemoglobin AS status, G6PD, ferritin, hemoglobin, and the red cell distribution width coefficient of variation (RDW-CV), and using stepwise Akaike information criterion procedures to select the best fit models.

## Results

### Participants

Of the 69 participants who consented and were screened, 64 completed the three consecutive sensor wears, including 29 children (4–17 years) and 35 young adults (18–26 years). Four failed screening (breastfeeding *n* = 1, A1c out of range *n* = 2, and T1D duration less than a year *n* = 1), and one did not complete the three sensor wears. Baseline characteristics of study completers are presented in Table 1. Two-thirds of the participants were patients at Mulago, and one-third at Nsambya. Thirty-nine percent of participants were poor or very poor as indicated in the table, with no electricity or solar power supply, piped water, flush toilets, or indoor kitchen. The majority of children were prepubertal; 52% were Tanner 1, 31% Tanner 2, and 17% Tanner 3 or greater. As expected, children were diagnosed at an earlier age and had shorter duration of diabetes than young adults. Baseline laboratory A1c and the total daily insulin dose were not significantly different between the children and young adults.

**Table 1.** Baseline Participant Characteristics (Mean ± SD)

	Child (4–17 years)	Young adult (18–26 years)	All ages (4–26 years)
*N*	29	35	64
Sex F/M (%F)	16/13 (55%)	15/20 (43%)	31/34 (48%)
Age (years)	12 ± 4	21 ± 3	17 ± 6
Age at type 1 diabetes (T1D) diagnosis (years)	8 ± 4	12 ± 5	10 ± 5
Duration T1D (years)	4 ± 3	8 ± 5	6 ± 5
Insulin NPH+reg/Mixtard/glargine+reg (*N*)	28/1/0	26/8/1	54/9/1
Units/kg/day insulin	0.9 ± 0.3	0.8 ± 0.3	0.8 ± 0.3
Baseline laboratory A1c (%)	10.9 ± 2.4	10.5 ± 2.0	10.7 ± 2.2
Baseline POC A1c (%)	11.1 ± 1.6	10.4 ± 2.1	10.7 ± 1.9
BMI (kg/m^2^)	NA	22.1 ± 3.6	NA
BMI (percentile)	18 ± 4	NA	NA
Socioeconomic status VP/P/LI/MHI (*N*)	4/6/7/12	5/10/7/13	9/16/14/25

All participants were Black.

NPH+Reg = multiple daily doses of NPH and regular insulin; Mixtard = twice daily premixed 70% NPH/30% regular insulin; glargine+reg = once daily glargine plus multiple daily injections of regular insulin (2–3); POC = point of care. Socioeconomic status was defined by housing circumstances and included 14% very poor (grass thatched/mud/wattle home, and no electricity/solar power supply, piped water, flush toilets, or indoor kitchen), 25% poor (home is not grass thatched/mud/wattle, but does not have electricity/solar power supply, piped water, flush toilets, or indoor kitchen), 21% low income (house has at least one but not all: electricity/solar power supply, piped water, flush toilets, indoor kitchen), and 40% high/middle income (house has all of the following: electricity/solar power supply, piped water, flush toilets, indoor kitchen).

### Metabolic characteristics

Sensor and A1c data are depicted in Table 2. The mean laboratory and POC A1c levels measured at the end of the third sensor were 10.7% ± 2.2% (94 ± 24 mmol/mol) and 10.7% ± 1.9% (94 ± 20 mmol/mol), respectively. Sensor data were collected for an average of 34 ± 4 days (range 31–41). Diabetes metabolic control, averaged across the three sensor wears, was in general very poor in comparison with established target goals.^17^ Mean glucose was 257 ± 57 mg/dL (14.3 ± 3.2 mmol/L). For the group, an average of only 27% ± 14% of time was spent in the target glucose range of 70–180 mg/dL (3.9–10.0 mmol/L), and only two of the 64 individuals spent at least 70% time in this range. On average, the group spent 68% ± 17% of time with glucose >180 mg/dL (10.0 mmol/L), and 48% ± 19% of time >250 mg/dL (13.9 mmol/L). Hypoglycemia was also common, with 55% of participants experiencing glucose levels <70 mg/dL (3.9 mmol/L) ≥ 4% of the time, and 42% of participants experiencing glucose levels <54 mg/dL (3.0 mmol/L) ≥1% of time. Average glucose CV was 44% ± 10% (range 23%–62%), and 80% of participants had a CV level greater than the recommended maximum level of 36%.^19^

**Table 2.** Final Metabolic Characteristics (Mean ± SD). Continuous Glucose Monitoring (CGM) Values were the Average of Three Consecutive Sensor Wears Over 31–41 Days (Average 34 ± 4 Days) with No More Than 48 H Between Sensor Wears. A1c was Measured in the Laboratory and As POC at the End the Three Sensor Wears. Time‐in‐Range Targets are as Recommended by the International Consensus on Time in Range^16^

	Child(4–17 years)	Young adult(18–26 years)	All ages(4–26 years)	Child vs. adult*P* value
*N*	29	35	64	
Average CGM glucose mg/dL (mmol/L)	270 ± 54 (15.0 ± 3.0)	247 ± 61 (13.7 ± 3.4)	257 ± 57 (14.3 ± 3.2)	0.128
Coefficient of variation %	44 ± 8	44 ± 10	44 ± 9	0.847
Laboratory A1c % (mmol/mol)	10.1 ± 1.8 (87 ± 20)	10.1 ± 2.2 (87 ± 24)	10.1 ± 2.0 (86 ± 21)	0.997
POC A1c % (mmol/mol)	10.7 ± 1.3 (94 ± 15)	10.0 ± 2.3 (86 ± 25)	10.3 ± 2.0 (89 ± 21)	0.130
Percent time glucose >180 mg/dL, 10.0 mmol/L (target <25%)	71 ± 14	65 ± 19	67 ± 16	0.155
Percent time glucose 70–180 mg/dL, 3.9–10.0 mmol/L (target >70%)	25 ± 12	30 ± 16	27 ± 14	0.171
Percent time glucose <70 mg/dL, 3.9 mmol/L (target <4%)	5 ± 4	5 ± 5	5 ± 4	0.388

As the CV increased beyond 36%, the percent time in hypoglycemia progressively increased while the MG was consistently above target. Consistent with prior findings,^19^ greater than 1% time spent below 54 mg/dL (3.0 mmol/L) was only observed at CV levels of 40% or greater (Spearman’s *r* = 0.73, *P* < 0.0001, Supplementary Fig. S2). The only significant difference in CGM parameters between children and adults was slightly less time spent with glucose <70 mg/dL (3.9 mmol/L) in children: 4% ± 3% versus 6% ± 6% (Table 2).

### A1c and MG

**FIG. 1.**
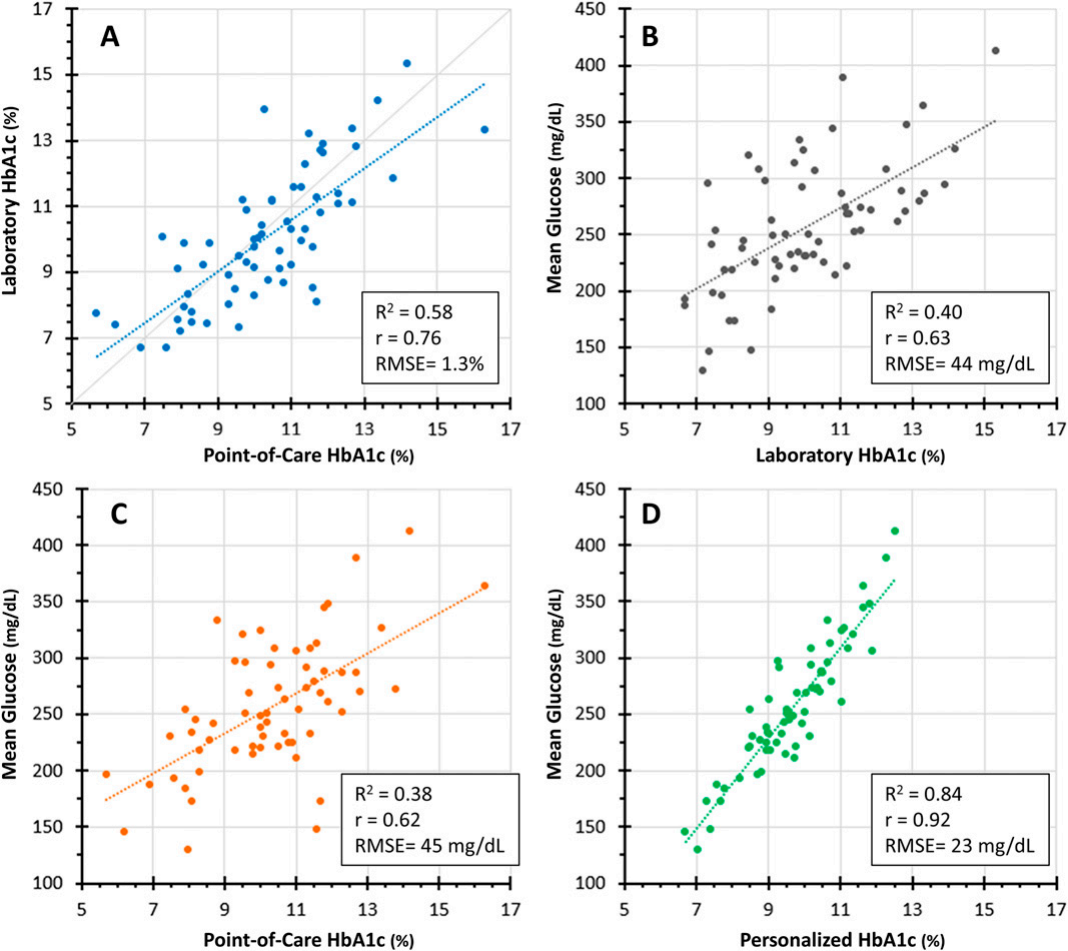
Hemoglobin A1c (A1c) and mean glucose (MG) data after three sensor wears over 31–41 days (average 34 ± 4 days). For any given point-of-care (POC) A1c, there were a wide range of simultaneously obtained laboratory A1c levels with no consistent pattern of one being higher or lower than the other (Fig. 1A). The A1c and MG were moderately correlated, whether A1c was measured in the laboratory (Fig. 1B) or POC (Fig. 1C), and the fitted regression lines had very similar intercept and slope no matter which A1c method was used. MG concentrations versus personalized A1c levels (pA1c)^8,12,18^ are shown in Figure 1D. Although there was considerably less variability compared with the relationship between MG and laboratory A1c, as shown in Figure 1B (*R*^2 ^= 0.84 vs. 0.40, *r* = 0.92 vs. 0.63), for a given pA1c level there were still a clinically significant range of MG concentrations, particularly for pA1c levels between 8.5% and 11% (69–97 mmol/mol), a span that represented the majority of participants. The error of the estimate for the pA1c, presented as the RMSE (root mean square error), demonstrates relative improvement in Figure 1D compared with Figure 1B, C.

POC A1c levels showed good correlation with simultaneously obtained laboratory A1c levels (*R*^2 ^= 0.58, *r* = 0.76), but for any given POC A1c there was a wide range of laboratory A1c levels with no consistent pattern of one being higher or lower than the other (Fig. 1A). The A1c and MG were moderately correlated, whether A1c was measured in the laboratory (*R*^2 ^= 0.40, *r* = 0.63, Fig. 1B) or by POC (*R*^2 ^= 0.38, *r* = 0.62, Fig. 1C), and the fitted regression lines had very similar intercepts and slopes no matter which A1c method was used.

Glycemic extremes were common, with many patients experiencing both marked hyper- and hypoglycemia, and this was not adequately reflected by the A1c levels. Fifty percent of participants had MG concentrations >250 mg/dL (13.9 mmol/L). Within this severely hyperglycemic group, mean A1c was 11.1% ± 1.9% (98 ± 21 mmol/mol), but there was marked variability with A1c levels ranging from 7.3% to 15.3% (56–144 mmol/mol). Thirty-eight percent of this group also spent more than 4% of time with glucose levels <70 mg/dL (3.9 mmol/L). Supplementary Figure S3 demonstrates the wide glycemic variability seen in individual subjects at the lower, middle, and upper end of the laboratory A1c spectrum, and emphasizes how much important clinical information is missed when relying on any measure of A1c or MG in this population.

### Personalized A1c

There was a substantially improved correlation between pA1c and MG (*R*^2^ = 0.84, *r* = 0.92 [Fig. 1D]) compared with laboratory A1c and MG [Fig. 1B]). However, despite this strong correlation, for a given pA1c level, there was still a clinically significant range of MG concentrations, particularly for pA1c levels between 8.5% and 11% (69–97 mmol/mol), an A1c span that included almost three-quarters of participants. It should be noted that the calculations for pA1c assume relatively steady glucose concentrations, which was not the case in this population.

### Relationship to ADAG and Bergenstal data

**FIG. 2.**
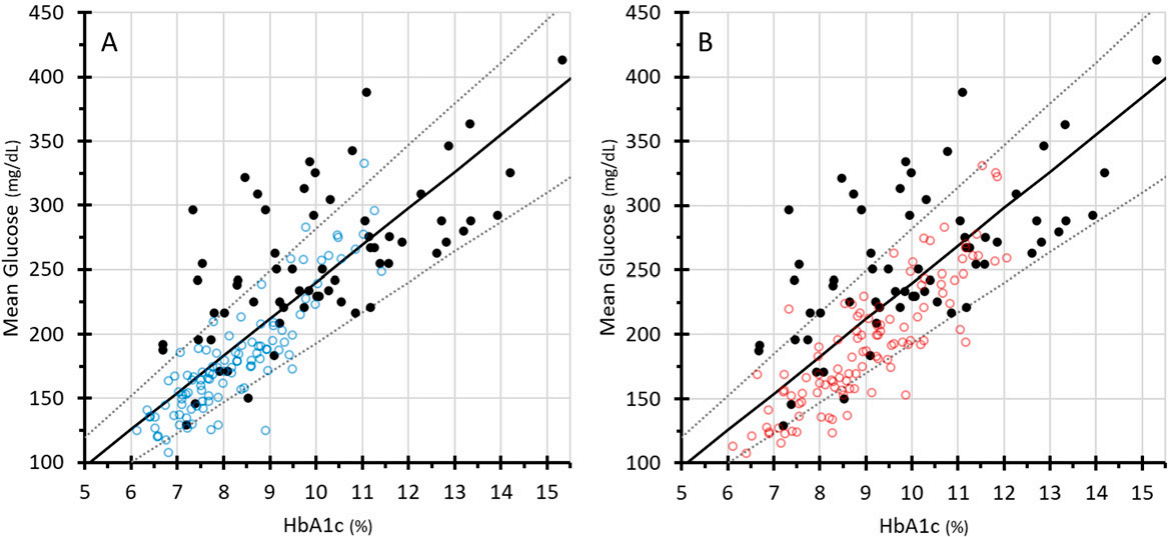
The black regression lines in both panels represent the mean and 95th percentile confidence intervals from ADAG (the A1c-Derived Average Glucose Study Group).^20^ The blue dots (non-Hispanic Whites, panel A) and the red dots (Non-Hispanic Blacks, panel B), represent the U.S. participants reported by Bergenstal et al.^3^ The black dots represent our Ugandan cohort. The Bergenstal data show modest discordance in general from the ADAG data, and racial differences in the A1c-MG relationship. The Ugandan A1c-MG data are discordant from both data sets. Hyperglycemia was much more prevalent in the Ugandan participants, with 50% of MG concentrations >250 mg/dL (13.9 mmol/L), and one-third of A1c levels >11% (97 mmol/mol). The Bergenstal data are publicly available at https://public.jaeb.org/datasets/diabetes.

The A1C-Derived Average Glucose Study Group (ADAG) reported the relationship between A1c and MG in 507 individuals with T1D, T2D, and no diabetes.^21^ Those with diabetes had to have stable glycemia and no conditions that might result in major glycemic changes. Figure 2 shows regression lines representing the mean and 95th percentile confidence intervals from ADAG, which have been widely used clinically. Superimposed on this are the U.S. non-Hispanic White (Fig. 2a, blue dots) and Black (Fig. 2B, red dots) participants reported by Bergenstal et al,^3^ as well as our Ugandan cohort (black dots). The Bergenstal data demonstrate racial differences in the A1c-MG relationship compared with ADAG data. The Ugandan A1c-MG data are discordant from both data sets. What is most striking is the overall worse metabolic control in the Ugandan participants, with a far greater number of them having MG levels >250 mg/dL (13.9 mmol/L) and A1c levels >11% (97 mmol/mol). In addition, the Ugandans exhibited a much wider range of A1c levels for any given MG compared with those in the other two data sets.

### Other factors that might influence A1c

Malaria blood smears for parasites were all negative, and peripheral smears and reticulocyte counts did not show signs of active hemolysis in any participant. Two participants (3%) were anemic with hemoglobin levels <11 g/dL and low ferritin levels. Hemoglobin electrophoresis showed normal hemoglobin AA in 87% of participants, while sickle-cell trait (hemoglobin AS) was found in 13%. Ten percent of participants had low G6PD levels. The covariates sex, ferritin, and hemoglobin were significant in regressing laboratory A1c or pA1c on MG, while all other demographic and clinical factors were nonsignificant. Adjusting for these three covariates did not change the conclusions on the associations between laboratory A1c or pA1c versus MG.

## Discussion

CGM is currently not available or affordable in sub-Saharan Africa, and diabetes care providers rely heavily on A1c measurements to guide insulin adjustment decisions. In a first of its kind study among East African children and youth with T1D, we used a brief period of CGM (two consecutive sensors) to calculate individual AGRs, which were subsequently used to personalize A1c measurements. We sought to determine if the pA1c would provide useful clinical information by more accurately reflecting MG than the measured A1c, and thus mitigate the need for chronic CGM use. Because POC is the most common A1c measurement in this setting, we assessed the A1c-MG relationship using both POC A1c and the more accurate laboratory-measured A1c. The pA1c did reduce variation in the A1c-MG relationship and was a better reflection of MG than measured A1c levels. However, no matter how accurately MG is estimated, it is still just an average and thus has limited clinical application when glycemic excursion is as wide and unpredictable as in our cohort. Thus, in this low/low-middle income setting, solely using A1c, pA1c, or other estimates of the A1c-MG relationship as a tool to guide insulin dosing would be misguided at best and potentially dangerous due to underlying hypo- and hyperglycemia levels that might be missed.

After publication of the Diabetes Control and Complications Trial, specific A1c targets were recommended for diabetes management. In 2017, Beck et al. suggested that while A1c provided important population-level data, it might not be suitable to guide the management of individual patients because of variability in the relationship between A1c and MG.^2^ The glucose management indicator (GMI), now included on standard CGM printouts, was developed to provide clinicians with the 10–14-day CGM-measured MG, converted into the more familiar A1c units.^4^ The GMI formula does not account for individual- or population-level variation in the A1c-MG relationship.

Xu et al. took this a step further, developing an individualized approach that involves calculation of an AGR (personal).^8,13,18^ Once determined, the AGR “corrects” future measured A1c levels, based on the individual’s characteristic glycation tendency, to more accurately reflect their MG concentration and thus to help optimize clinical decision-making. This approach appears to be promising in individuals with diabetes living in high-income countries such as the United States, from whom AGR calculations were derived,^3,8^ and where overall metabolic control has been positively influenced over the years by the availability of analog insulin and the capacity for frequent glucose monitoring to guide therapy.^22^ Our data suggest that its utility is limited, however, in populations with extreme glycemic instability.

The relationship between MG and A1c is well known to vary between individuals and populations. A twin study demonstrated that 62% of the population variance in A1c may be genetic.^23^ Proposed genetic mechanisms involve factors influencing the glycation of hemoglobin, RBC lifespan, or RBC GLUT1 glucose uptake.^4,18,24^, Racial differences have been observed, with African Americans reported to have lower MG levels than Whites for a given A1c level.^3,5,7–9,25^ In contrast, this young East African cohort demonstrated higher MG levels. One striking difference between this and other studies is that 50% of the Ugandan participants had MG levels >250 mg/dL (13.9 mmol/L); in the Bergenstal study, <20% of participants were this hyperglycemic.^3^ Interestingly, the A1c-MG relationship in Ugandan participants whose MG was <250 mg/dL (13.9 mmol/L) more closely approximated the Bergenstal data for African Americans,^3^ and their pA1c-MG relationship more closely fit the regression line (Fig. 1D), suggesting that pA1c was a more reliable estimate for patients with better metabolic control and lower MG levels. It is possible that genetic/racial contributions to the A1c-MG relationship, if present, may have been obscured by the magnitude of poor glycemic control in our population.

The young age of our participants (4–16 years) might be relevant to our findings, since MG levels for a given A1c are known to be higher in youth compared with adults.^8^ Clinical factors that affect RBC lifespan were considered, but did not appear to play a role. In U.S. studies, neither RBC indices^26^ nor iron status^27^ explained racial difference in the A1c-MG relationship; similarly, we were not able to detect an influence of RBC indices, iron status, anemia, malaria, sickle-cell trait, or G6PD deficiency. Our numbers may have been too small to detect subtle effects related to these or other unknown clinical factors.

A secondary analysis of data from our previously published pilot study in a demographically similar group of Kenyan and Ugandan youth found that MG concentrations were lower than expected for A1c levels^9,12^—the opposite of what we found in the current study. The most important difference between these studies is that hypoglycemia was much more frequent and more severe in the pilot. The protracted hypoglycemia experienced by patients across all A1c levels in that study may have contributed to lower-than-expected MG concentrations. We attribute the reduction in hypoglycemia over the approximately 5 years between the pilot and the current study to be due, at least in part, to better patient education, and, in particular, to the phasing out of fixed ratio insulin Mixtard (70% NPH, 30% regular insulin). This appears to have substantially reduced hypoglycemia but not hyperglycemia.

Why are glucose levels so unstable in this population? In much of the world, insulin analogs appear to have reduced both glucose variability and the risk of hypoglycemia.^22^ Less expensive human insulins such as NPH and regular are usually all that is available in East Africa, and these may peak at inconvenient times that have little relationship to food intake or activity. Access to between-meal food is often difficult. For some patients, this is related to food insecurity, but even for well-nourished patients, food is generally not readily available between designated meal or snack times. The vending machines, convenience stores, and processed/packaged snack foods and beverages that are ubiquitous in the United Sates are not part of that culture. Also, children and youth in Uganda are often much more active than their North American and European counterparts, with physically demanding jobs or chores at home, and long walks to and from school. East African patients have anecdotally described reducing or omitting insulin for fear of hypoglycemia. T1D management may also be complicated by higher risk of infection and intercurrent illness, poverty, and lack of patient, family, and community education.

How can health care providers in East Africa more safely manage both hyper- and hypoglycemia in their patients with T1D? Our data demonstrate that using A1c as a surrogate for MG does not provide sufficient information for safe insulin adjustment, whether A1C is measured in the laboratory or by POC, or when it is personalized based on individual characteristics. Neither A1c nor MG measures can replace the information that is available from CGM systems about the range of glycemic excursion, glucose patterns throughout the day and night, and the percent time at various glucose ranges. This information is particularly important given the challenges of T1D management in this setting. At a minimum, intermittent CGM, perhaps once every 3–6 months, might help improve glycemic control and reduce hypoglycemia by providing a period of comprehensive glucose data to guide appropriate insulin adjustment.

This study has several strengths. The protocol, which included training and monitoring by experienced research staff from UMN, was performed at two major pediatric diabetes centers in Uganda with no major protocol deviations, and study performance met the all-regulatory requirements. The sensors were well tolerated by patients. It is the first study to assess a steady-state model of a pA1c approach in an African population and in a low/low-middle income setting. There are also limitations. The participant number was relatively small, although it was sufficiently powered to detect correlations between A1c and MG. CGM readings were not collected over the full expected RBC lifespan; in contrast, the study used to develop the pA1c level had about 3 months of sensor data.^8,13^ It is possible that a longer period of CGM would have led to more stable glycemia, although data from an ongoing study suggest that even 6 months of blinded sensor wear in this population does not substantially reduce the CV. Imprecision in A1c assays can be a source of error^28^; we were not able to attain quality control information such as the MARD (mean absolute relative difference) or MAD (mean absolute difference) relative to the glucose level for either the POC or the laboratory A1c methods. Our observations about the relationship between A1c and MG in this young Ugandan cohort may not be generalizable to other populations, other settings, or in older age groups.

## Conclusions

We report significant discordance in the A1c-MG relationship in a young East African population with T1D, whether A1c was measured in the laboratory or by POC. While calculating a pA1c allowed a more accurate estimation of MG, it did not provide reliable guidance for insulin adjustment in this population with extreme glycemic instability. CGM systems have largely replaced SMBG in high-income settings and the American Diabetes Association considers them to be standard of care for T1D.^29^ These systems are currently unaffordable in lower income settings, but our data suggest that it is essential that a way be found to make them at least periodically affordable and available.

## Data Availability

Some or all datasets generated during and/or analyzed during the current study are not publicly available but are available from the corresponding author on reasonable request.
