# Supplementary material for: Personalized Hemoglobin A1c Shows Better Correlation with Mean Glucose than Laboratory Hemoglobin A1c in Ugandan Youth with Type 1 Diabetes, but Mean Glucose Is Not Clinically Useful in This Population Due to Extreme Glucose Variability
Source: Diabetes Technol Ther. 2025 Jul 29;27(8):641–50. doi: 10.1089/dia.2024.0537 (PMC12955361; doi:10.1089/dia.2024.0537)

Supplemental Figure 1. Study Design. Three consecutive Libre Pro sensors were placed with no more than 48 hours between sensor wears. The mean glucose over the first two sensor wears and the laboratory A1c at the end of the second sensor wear were used to calculate the apparent glycation ratio (AGR). Once determined, the AGR "corrects" future measured A1c levels, based on that individual's characteristic glycation tendency, so that the measured A1c more accurately reflects their mean glucose concentration[8,12,18]. The mean glucose over the three sensor wears was calculated (reported as MG throughout the manuscript), and the AGR was applied to the final laboratory A1c level to calculate a personalized A1c level (pA1c).

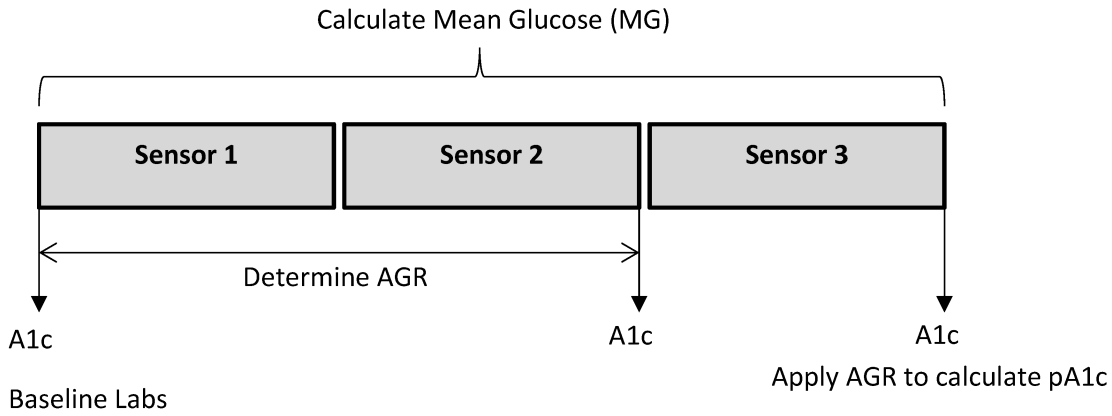

Supplement: Supplementary Figure S1 [file dia.2024.0537_Supplementary_Figure_S1.pdf]
