# Supplementary material for: Personalized Hemoglobin A1c Shows Better Correlation with Mean Glucose than Laboratory Hemoglobin A1c in Ugandan Youth with Type 1 Diabetes, but Mean Glucose Is Not Clinically Useful in This Population Due to Extreme Glucose Variability
Source: Diabetes Technol Ther. 2025 Jul 29;27(8):641–50. doi: 10.1089/dia.2024.0537 (PMC12955361; doi:10.1089/dia.2024.0537)

Supplemental Figure 2. Percent time glucose <54 mg/dl (3.0 mmol/L) (Panel A) and percent time <70 mg/dl (3.9 mmol/L) (Panel B) versus the coefficient of variation. Dashed lines are theorectical values according to an underlying gamma-shaped glucose distribtion at different mean glucose levels ranging from 150 to 450 mg/dL.

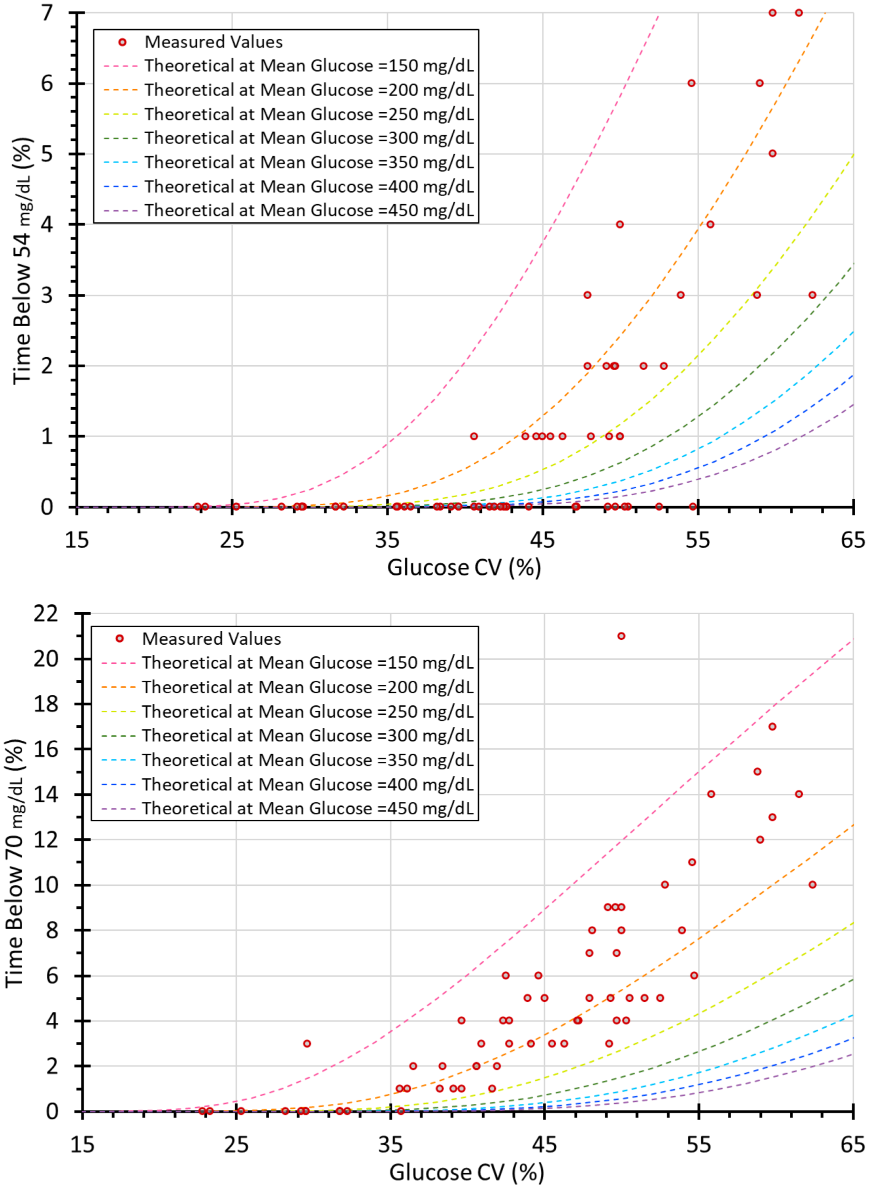

Supplement: Supplementary Figure S2 [file dia.2024.0537_Supplementary_Figure_S2.pdf]
