# Supplementary material for: Personalized Hemoglobin A1c Shows Better Correlation with Mean Glucose than Laboratory Hemoglobin A1c in Ugandan Youth with Type 1 Diabetes, but Mean Glucose Is Not Clinically Useful in This Population Due to Extreme Glucose Variability
Source: Diabetes Technol Ther. 2025 Jul 29;27(8):641–50. doi: 10.1089/dia.2024.0537 (PMC12955361; doi:10.1089/dia.2024.0537)

Supplemental Figure 3. CGM data demonstrate the difficulties in relying on either laboratory A1c (LA1c) or point-of-care A1c (POC) levels for clinical decision making in this population, because of wide variation in mean glucose levels and the times in various glucose ranges for any given A1c level. To illustrate this with clinical examples, we selected four participants from each of three different laboratory A1c tiers: the four indivudals who were closest to the cohort baseline mean of 10.7% (93 mmol/ml), four at the lower end of the LA1c range, and four at the upper end. No measure of A1c (laboratory, POC or personalized) captured the range of glycemia or the dangerously high percent time in hypoglycemia found in many patients, limiting the usefulness of A1c in this setting and emphasizing the importance of CGM data when considering insuln adjustment. A1c values are presented as % (mmol/mol). MG=mean CGM glucose mg/dL (mmol/L), pA1c=personalized HbA1c[8,12,18]; TIL2Hypo (time in Level 2 hypoglycemia) = % time glucose levels <54 mg/dL (3.0 mmol/L), TIL1 Hypo (time in Level 1 hypoglycemia) = % time glucose levels 54-69 mg/dL (3.0-3.8 mmol/L), TIR = percent time glucose levels 70-180 mg/dL (3.9-10.0 mmol/L), TIL1Hyper (time in Level 1 hyperglycemia) = percent time glucose levels 181-250 mg/dL (10.1-13.9 mmol/L), and TIL2Hyper (time in Level 2 hyperglycemia) = percent time glucose levels >250 mg/dL (>13.9 mmol/L). CGM-estimated measures are the average of three consecutive sensor wears and A1c was measured at the end of the third sensor.

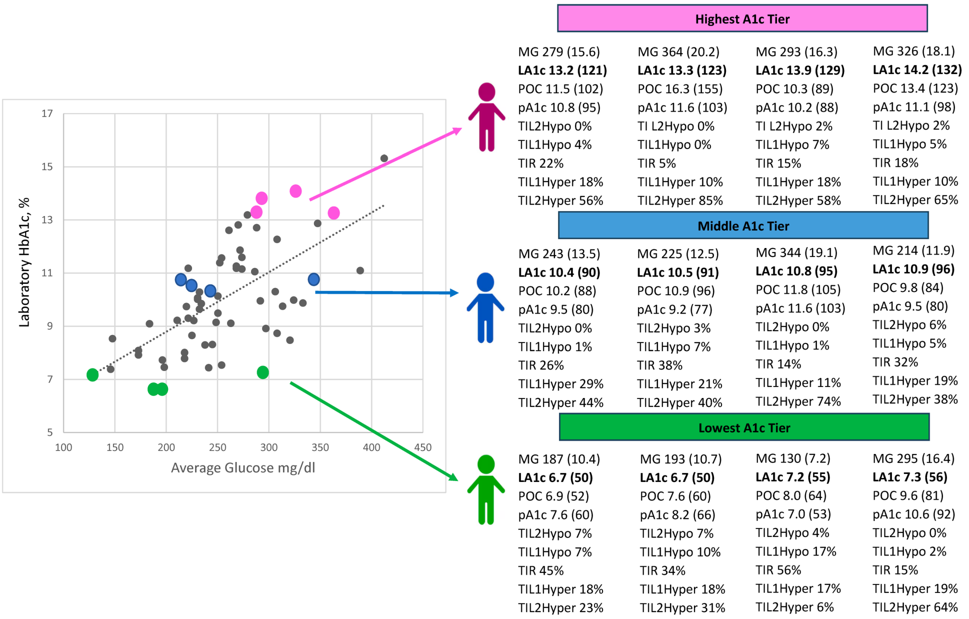

Supplement: Supplementary Figure S3 [file dia.2024.0537_Supplementary_Figure_S3.pdf]
